# Frizzled 7 and PIP_2_ binding by syntenin PDZ2 domain supports Frizzled 7 trafficking and signalling

**DOI:** 10.1038/ncomms12101

**Published:** 2016-07-08

**Authors:** Antonio Luis Egea-Jimenez, Rodrigo Gallardo, Abel Garcia-Pino, Ylva Ivarsson, Anna Maria Wawrzyniak, Rudra Kashyap, Remy Loris, Joost Schymkowitz, Frederic Rousseau, Pascale Zimmermann

**Affiliations:** 1 Centre de Recherche en Cancérologie de Marseille (CRCM), Inserm, U1068-CNRS UMR7258, Aix-Marseille Université, Institut Paoli-Calmettes, 13009 Marseille, France; 2 Department of Human Genetics, KU Leuven, ON1 Herestraat 49 Box 602, B-3000 Leuven, Belgium; 3 VIB Switch Laboratory, Department of Molecular Cellular and Molecular Medicine, VIB-KU Leuven, B-3000 Leuven, Belgium; 4 Structural Biology Brussels, Deptartment of Biotechnology (DBIT), Vrije Universiteit Brussel and Molecular Recognition Unit, Structural Biology Research Center, VIB, Pleinlaan 2, B-1050 Brussel, Belgium

## Abstract

PDZ domain-containing proteins work as intracellular scaffolds to control spatio-temporal aspects of cell signalling. This function is supported by the ability of their PDZ domains to bind other proteins such as receptors, but also phosphoinositide lipids important for membrane trafficking. Here we report a crystal structure of the syntenin PDZ tandem in complex with the carboxy-terminal fragment of Frizzled 7 and phosphatidylinositol 4,5-bisphosphate (PIP_2_). The crystal structure reveals a tripartite interaction formed via the second PDZ domain of syntenin. Biophysical and biochemical experiments establish co-operative binding of the tripartite complex and identify residues crucial for membrane PIP_2_-specific recognition. Experiments with cells support the importance of the syntenin–PIP_2_ interaction for plasma membrane targeting of Frizzled 7 and c-jun phosphorylation. This study contributes to our understanding of the biology of PDZ proteins as key players in membrane compartmentalization and dynamics.

## Abstract (web summary)

PDZ domain-containing proteins are known to function as intracellular scaffolds. Here, Egea-Jimenez *et al*. report the structure of the tandem PDZ domains of syntenin in complex with a Frizzled 7 peptide and PIP2, show that the ligands bind to syntenin cooperatively and illustrate the role of the complex for Frizzled 7 function.

## Graphical abstract

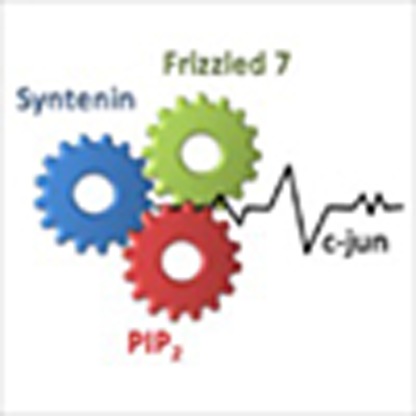

PDZ domains are among the most common interaction modules in the human proteome, with ∼270 domains imbedded in >150 proteins^1^. Their name is derived from the first three proteins in which these domains were found, namely the post-synaptic protein PSD-95, the *Drosophila* discs large protein and the tight junction protein ZO1. PDZ domains consist of 80–90 amino-acid residues that fold into a globular shape consisting of six β-strands arranged in a β-sandwich and two α-helices capping each end with a βββαββαβ topology. They are found in cytosolic scaffold proteins that often contain multiple PDZ domains and other protein-binding modules. PDZ proteins are involved in a wide range of cellular processes, including the regulation of receptor tyrosine kinase signalling, the establishment and the maintenance of cell polarity, the control of protein trafficking and the co-ordination of synaptic events^2^,^3^,^4^,^5^. Most binding events that are mediated by PDZ domain are due to the interaction of PDZ domains with the C-terminal end of their target proteins. In these cases, the peptide ligand docks into an elongated groove between the second β strand and the second α helix of the PDZ domain. However, PDZ domains are also able to bind internal peptide sequences, phospholipids and to mediate protein dimerization^6^,^7^,^8^,^9^.

Syntenin is a small protein containing two closely linked PDZ domains (PDZ tandem). It was originally identified as a syndecan PDZ-binding protein^10^,^11^. Yet, the binding repertoire of syntenin PDZ domains is promiscuous and broad, as is the case for other PDZ proteins^12^,^13^,^14^,^15^. The PDZ domains of syntenin also interact for example with certain Frizzled receptors (for example, Frizzled 3, -7 and -8). Syntenin was reported to control non-canonical Wnt signalling and early polarized movements in *Xenopus* and zebrafish^16^,^17^. Importantly, syntenin PDZ domains also interact with phosphatidylinositol 4,5-bisphosphate (PIP_2_)^18^ and this interaction was shown to support the recycling of syndecans and potentially their heparan sulfate cargo (growth factors and adhesion molecules) from endosomal compartments to the plasma membrane with important consequences for cell behaviour and in particular cell spreading^19^. These original findings were followed by a number of reports highlighting the link between PDZ domains and phosphoinositides, more recently other membrane lipids like cholesterol^20^,^21^,^22^,^23^,^24^,^25^,^26^,^27^.

Phosphoinositides are phosphorylated derivatives of phosphatidylinositol (PI). They contain two long hydrophobic fatty acyl chains linked to a glycerol group that is coupled via a phosphodiester bond to the phosphorylated inositol group. The inositol group can be phosphorylated at positions 3, 4 and/or 5 of its inositol ring generating seven phosphoinositide species. The cellular distribution of phosphoinositides is tightly regulated by a network of kinases and phosphatases that are in turn controlled by signalling. Each of the seven phosphoinositides shows a specific subcellular enrichment. For example PIP_2_ predominates at the plasma membrane, whereas phosphatidylinositol 3-phosphate is enriched in early endosomes. Phosphoinositides are involved in nearly all aspects of cell biology, including membrane trafficking, cytoskeleton remodelling, regulation of ion channels and transporters, gene transcription, RNA editing and cell cycle progression^28^,^29^. Clearly, at least a subgroup of PDZ domains interacts with phosphoinositides. Other lipids can assist phosphoinositide interactions, and peptide binding can either compete or cooperate with phosphoinositide binding depending on the combination of ligands^7^,^20^,^21^,^22^. Despite these findings, crystallographic data addressing PDZ–phosphoinositide interactions are currently lacking, precluding a precise molecular understanding of the phenomenon.

In this paper, we report the crystal structure of the syntenin PDZ tandem in complex with the carboxy-terminal fragment of Frizzled 7 and PIP_2_. We observe a tripartite interaction that engages the second PDZ domain (PDZ2) of syntenin. We validate the structure through a combination of biophysical and biochemical experiments that support a tripartite cooperative binding of Frizzled 7 and PIP_2_ by the PDZ2 domain. We determine that asparagine 215, and lysines 214 and 250 in the PDZ2 domain, and lysine 569 in Frizzled 7 (further referred to as lysine-5 in the carboxy-terminal fragment) are essential for membrane PIP_2_-specific recognition. Finally, we establish the importance of syntenin–PIP_2_ interaction and of these residues for the plasma membrane targeting of Frizzled 7, and for Frizzled 7 signalling in cells.

## Results

### Frizzled 7 and PIP_2_ simultaneously bind to syntenin PDZ2

**Figure 1:**
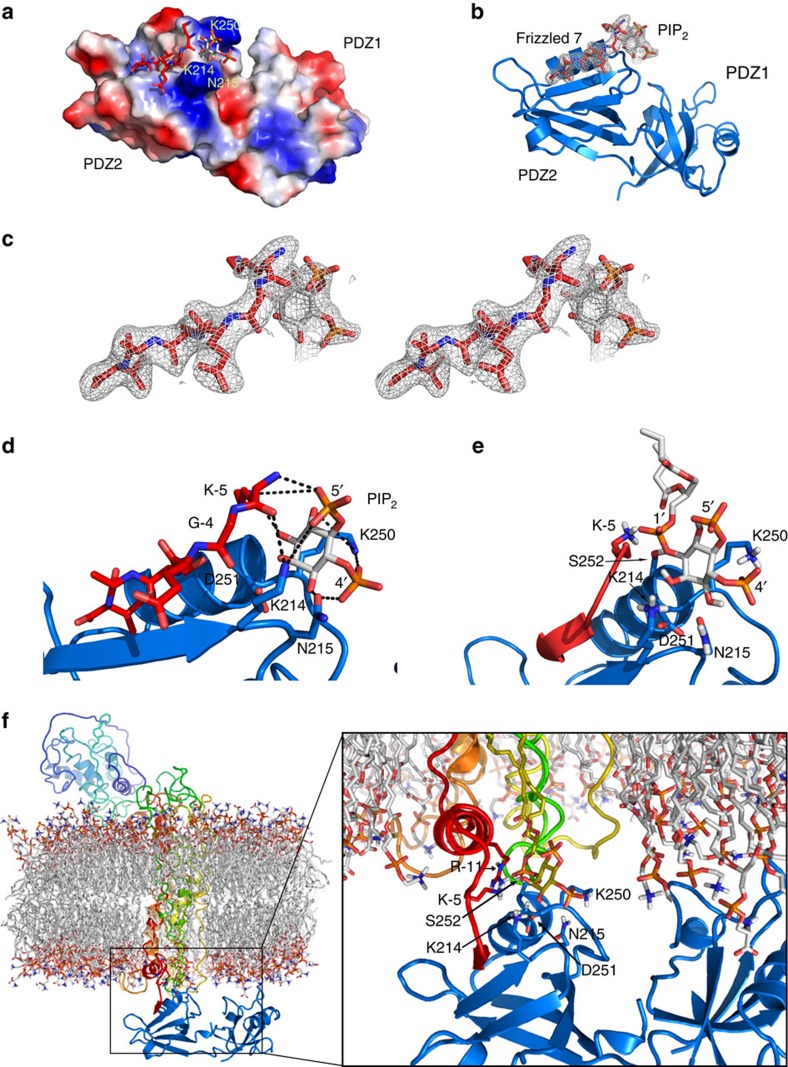
Model of the ternary complex of syntenin PDZ tandem-Frizzled 7-PIP_2_. (**a**) Surface representation of the PDZ tandem of syntenin coloured according to electrostatic potential, highlighting the slightly basic character of the PIP_2_ binding site. Red is negative, blue is positive and white is neutral. The bound Frizzled 7 peptide and the PIP_2_ head group are shown in red and CPK sticks, respectively. (**b**) Frizzled 7 peptide binds in the peptide-binding groove of the PDZ2 domain of the PDZ tandem (blue ribbon) by canonical β-addition. The C4-PIP_2_ molecule binds in a pocket next to the end of the peptide-binding groove. The wire mesh (light grey) represents the electron density map (2*F*_c_−*F*_o_, *σ*=1) of the hexapeptide and C4-PIP_2_. (**c**) Stereo view of the electron density maps presented in **c**. (**d**) Detailed view of the PIP_2_-binding site. The residues from the syntenin PDZ2 and from the Frizzled 7 hexapeptide that form the PIP_2_-binding site are indicated. (**e**) Snapshot after simulated annealing energy minimization of the complex between the syntenin PDZ tandem, Frizzled 7 C-terminal hexapeptide and full C4-PIP_2_. The PDZ tandem and Frizzled 7 are represented as cartoon in blue and red colours, respectively. C4-PIP_2_ is represented as sticks in CPK colours. (**f**) Model of the PDZ tandem of syntenin interacting with the inner leaflet of the cell membrane through interactions with membrane-inserted Frizzled 7 and PIP_2_ as observed in the crystal structure. The PDZ tandem is represented as cartoon in blue colour with bound PIP_2_ represented as sticks with carbons in yellow and phosphates in CPK colours. Frizzled 7 is represented as cartoon in rainbow colours. The bound C-terminal end of Frizzled 7 is in red colour. The membrane lipids are represented as sticks with carbons in white colour and CPK for the headgroups. The side chains of the amino acids that form the PIP_2_-binding pocket are labelled and shown. The image corresponds to a snapshot after 20 ps of molecular dynamic simulation using Amber03 force field as provided in Yasara structure software.

To determine the molecular basis of Frizzled 7 and PIP_2_ binding by the PDZ domains of syntenin, we purified various constructs of syntenin (amino acids 107–275; 108–275; 113–273; 113–270; 107–192; 108–192; and 196–275) and tried to co-crystalize various combinations of bipartite and tripartite complexes. We succeeded in co-crystallizing the PDZ tandem of syntenin (amino acids 107–275) in the presence of the C-terminal hexapeptide of Frizzled 7 (KGETAV) and L-alpha-phosphatidyl-(1,2-dibutanoyl)-D-myo-inositol (C4-PIP_2_). The structure (Table 1) revealed that one Frizzled 7 peptide and one C4-PIP_2_ molecule bind simultaneously to the PDZ2 domain (Fig. 1a). The Frizzled 7 hexapeptide binds into the peptide-binding groove by canonical β-addition (Fig. 1b). The C4-PIP_2_ molecule (Fig. 1c) binds immediately adjacent to the peptide into a positively charged pocket allocated at the exit of the peptide-binding grove (Fig. 1a). Only the inositol ring, and phosphates 4 and 5 of C4-PIP_2_ show clear electron density and were modelled (Fig. 1c). The backbone and side chains of residues K214, N215, K250 and D251 together with K-5 from Frizzled 7 hexapeptide form the binding site of C4-PIP_2_ (Fig. 1d). The side chain of K214 interacts with the backbone of the Frizzled 7 hexapeptide, as well as the 5′ phosphate of the inositol ring. The side chain of N215 provides additional contacts with the inositol ring by hydrogen bonding the OH group at position 3′ and with the phosphate at position 4′. The backbone and amine group of K-5 from Frizzled 7 hexapeptide also contribute to the binding interface. The hydrocarbon portion of the side chain of K250 establishes van der Waals interactions with the inositol ring, whereas it’s amine group interacts with the 4′ phosphate group of PIP_2_ and further stabilizes the ligand (Fig. 1d). In addition, D251 provides crucial support in positioning the loop containing K214 and N215, by hydrogen bonding the peptide backbone (Fig. 1d).

To assess how the different components assemble together, we model the parts of C4-PIP_2_ not visible in the electron density. We completed the complex and performed an energy minimization through simulated annealing using Yasara^30^. After the simulated annealing, we observed the head group of C4-PIP_2_ completed with a phosphate group at 1′ is stably bound. By contrast, the aliphatic chains of the lipid remain detached from the surface of the PDZ tandem and oriented towards the bulk solvent, as expected from the lack of electron density (Fig. 1e).

We further modelled the PDZ tandem docked to a composite lipid membrane through the interactions with membrane-inserted Frizzled 7 and PIP_2_ as described on our crystallographic structure and submitted the complex to a molecular dynamics simulation using the Amber03 force field^31^, as implemented in Yasara. A snapshot after 20 ps of molecular dynamic simulation depicts how the PDZ tandem may interact with the membrane and how additional residues, such as R563 (or R-11) in Frizzled 7 and S252 in PDZ tandem, could stabilize the interaction with the phospholipid (Fig. 1f).

### Frizzled 7, PIP_2_ and syntenin PDZ 2 co-operative binding

**Figure 2:**
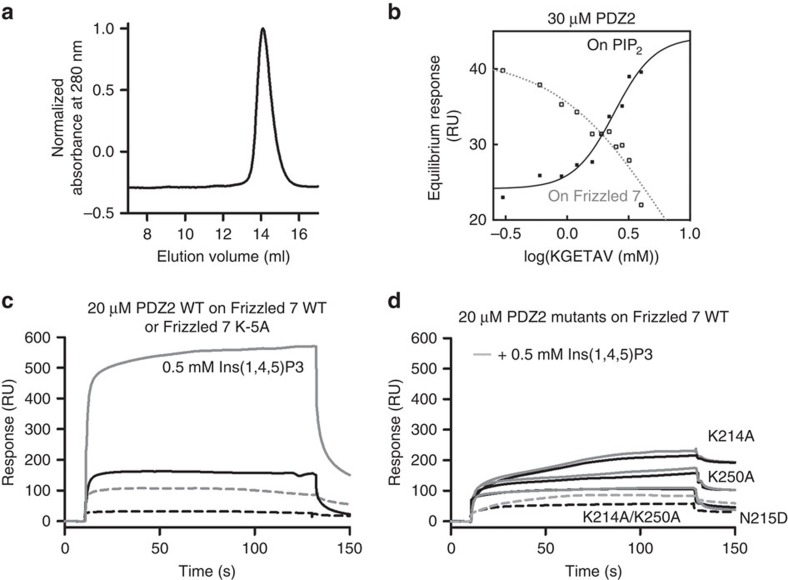
The PDZ2 domain of syntenin is a monomer that interacts with Frizzled 7 and PIP_2_ in a co-operative manner. (**a**) The elution profile of recombinant PDZ2 from an analytical size-exclusion column indicates that the polypeptide strictly behaves as a monomer (14 ml elution volume corresponding to ∼10 kDa). (**b**) Syntenin PDZ2 wild type (30 μM) was incubated with a range of 6mer Frizzled 7 peptide (KGETAV) concentrations (0–4 mM) before being perfused over immobilized PIP_2_ (black line and or filled circles) or 25mer Frizzed 7 peptide (dotted line and open circles) on BIAcore streptavidin (SA) chip. The equilibrium responses are plotted against the logarithm of the peptide concentration and the data fitted to the equation for log (agonist) versus response. The Hill coefficients for the co-operative binding between the KGETAV peptide in the solution and the PIP_2_ immobilized on the sensor chip was 2.6. (**c**) Blank-subtracted SPR sensorgrams. Binding of PDZ2 wild type (20 μM) over immobilized Frizzled 7 wild-type 19mer (black continuous line) or Frizzled 7 K-5A 19mer (black dotted line). A fixed concentration of 0.5 mM Ins(1,4,5)P3 (PIP_2_ head group) was pre-incubated with PDZ2 wild type (20 μM) before the protein was perfused over immobilized Frizzled 7 wild-type 19mer (grey continuous line) or Frizzled 7 K-5A 19mer (grey dotted line). (**d**) Blank-subtracted SPR sensorgrams. Interactions of PDZ2 single mutants (black continuous lines, mutations as indicate on top) or PDZ2 double mutant (black dotted line; 20 μM) with immobilized Frizzled 7 wild-type 19mer. A fixed concentration of 0.5 mM Ins(1,4,5)P3 (PIP_2_ head group) was pre-incubated with PDZ2 single mutants (grey continuous lines) or PDZ2 double mutant (grey dotted line; 20 μM) before perfusion over immobilized Frizzled 7 wild-type 19mer. RU, response units. Supplementary Fig. 1 illustrates SPR sensorgrams with various concentrations of proteins and equilibrium binding isotherms. Supplementary Fig. 2 shows that the secondary structure and the soluble state of PDZ2 wild type and double mutant are similar.

To further address the PDZ2–Frizzled 7–PIP_2_ interaction, we performed a set of surface plasmon resonance (SPR) experiments where purified recombinant syntenin PDZ2 domain (residues 196–275) was injected over immobilized biotinylated PIP_2_ or the 19 amino acids C-terminal peptide from Frizzled 7 (SWRRFYHRLSHSSKGETAV). We used a longer Frizzled 7 peptide for these experiments to ensure that the C-terminal PDZ-binding motif was far enough from the surface to be free to interact with the analyte. The isolated PDZ2 domain, which behaves as a monomer in solution (Fig. 2a), was used instead of the PDZ1–PDZ2 tandem to facilitate the interpretation of the data. To quantify the degree of co-operativity between ligands, we perfused a fixed concentration of PDZ2, pre-incubated with increasing concentrations of the C-terminal hexapeptide of Frizzled 7 (KGETAV) over immobilized PIP_2_. We observe a classical sigmoidal saturation curve with a Hill’s slope of 2.6 (Fig. 2b). In parallel, the PDZ2 domain was perfused over the immobilized Frizzled 7 peptide and as expected, we observed competition (Fig. 2b). We determined the apparent affinities of syntenin PDZ2 for Frizzled 7 in the absence of presence of IP_3_ (the head group of PIP_2_) by equilibrium titration experiments. The sensorgrams were corrected by subtracting signals obtained on blank surface and the observed equilibrium responses were plotted as a function of protein concentration (Supplementary Fig. 1a,e). The presence of 0.5 mM IP_3_ increases the apparent affinity of syntenin PDZ2 for Frizzled 7 (Fig. 2c; Supplementary Fig. 1a) by five-fold (from 32±3 μM to 5±2 μM; Supplementary Fig. 1a,e). In contrast, IP_3_ has no effect when syntenin PDZ2 is perfused over a mutant of Frizzled 7 (K-5A), in which the lysine at position 5 (implicated in PIP_2_ binding) is replaced by an alanine (Fig. 2c; Supplementary Fig. 1a), thus supporting the importance of this lysine for PIP_2_ binding.

To further challenge our structural model, we created a series of single mutants (K214A, N215D and K250A) and a double mutant (K214A/K250A) of PDZ2. These residues form the PIP_2_-binding site according to our structure, and support the simultaneous binding of Frizzled 7 peptide and PIP_2_ (Fig. 1c). The single mutations do not markedly affect Frizzled 7 interaction (compare Fig. 2c with Fig. 2d and Supplementary Fig. 1a with Supplementary Fig. 1d), but disrupt the cooperative binding of peptide and lipid (Fig. 2c,d). The double mutation, although it does not affect the overall structure of the PDZ2 domain (Supplementary Fig. 2a–c), affects the interaction with Frizzled 7 and also disrupts the cooperative binding of peptide and lipid (Fig. 2d, and Supplementary Fig. 1b,e). We also compared, in SPR experiments, the binding of syntenin PDZ2 to immobilized Frizzled 7 wild-type and Frizzled 7 K-5A mutant (SWRRFYHRLSHSS**A**GETAV). The presence of IP_3_ improves binding, but unfortunately the Frizzled 7 mutation decreases syntenin PDZ2 interaction, compromising a clear interpretation (Fig. 2c; Supplementary Fig. 1c).

**Figure 3:**
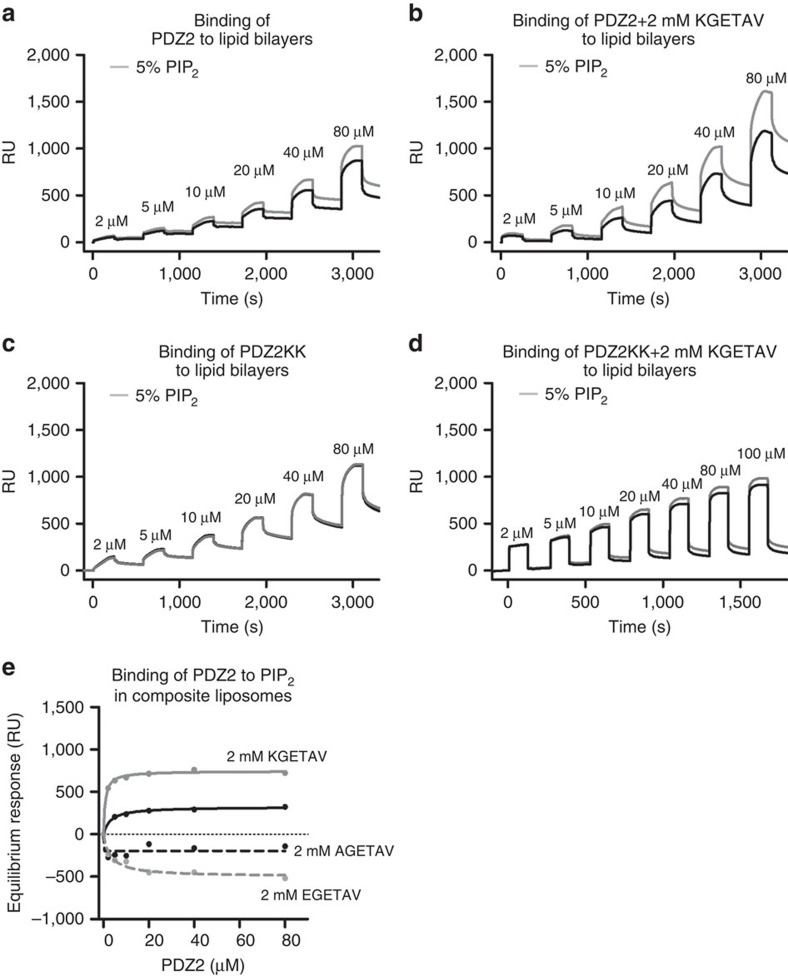
Frizzled 7 peptide enhances the interaction of syntenin PDZ2 with PIP_2_-containing composite liposomes. Sensorgrams illustrating the binding of increasing concentrations of PDZ2 wild-type (**a**,**b**) or PDZ2 mutant K214A/K250A (**c**,**d**) to liposomes containing 30% PC/40% PE/20% PS and either 10% PI (black line) or 5% PI and 5% PIP_2_ (grey line) in the absence (**a**,**c**) or in the presence (**b**,**d**) of 2 mM of KGETAV Frizzled 7 peptide. (**e**) Signals obtained at equilibrium using 5% PIP_2_-containing liposomes and PDZ2 wild type in the absence (black continuous line) or in the presence of 2 mM of Frizzled 7 peptides (KGETAV, grey continuous line; AGETAV, black dotted line; EGETAV, grey dotted line) were subtracted for association to 10% PI liposomes and plotted as a function of protein concentration. Note that solely the wild-type peptide (KGETAV) enhances PDZ2 interaction with PIP_2_ embedded in liposomes. The data show one experiment representative of three independent experiments done in duplicate. RU, response units. Supplementary Fig. 3 illustrates that the enhancement of PDZ2 interaction with PIP_2_ embedded in liposomes in the presence of Frizzled 7 peptide is lost when the PDZ2 carries K214A, K250A or N215D mutations. It also illustrates that PDZ1 in isolation strongly interacts with liposomes, but not with PIP_2_ embedded in liposomes, even in the presence of Frizzled 7 peptide.

To validate the above described observation in a biochemical experimental setting that better mimics the native cellular context of the protein, we performed experiments using PIP_2_ embedded in composite liposomes. The binding of wild-type and mutant PDZ2 to PIP_2_ was characterized using liposomes containing 30% phosphatidylcholine, 40% phosphatidylethanolamine, 20% phosphatidylserine and either 10% PI (as a control) or 5% PI, and 5% PIP_2_. Such composite liposomes reflect the physiological phospholipid composition of the inner plasma membrane leaflet^32^. For these SPR experiments, we used single-cycle kinetics^33^. This method consists of injecting the analyte at increasing concentrations, without regeneration steps between each sample injections to avoid the liposome being damaged (Fig. 3).

Wild-type PDZ2 binds to 5% PIP_2_-containing liposomes with a dissociation constant of 15±3 μM (after correction for ‘background association’ on 10% PI liposomes; Fig. 3a,e). When the PDZ2 wild type was pre-incubated with 2 mM of Frizzled 7 peptide (Fig. 3b,e), the dissociation constant dropped to 7±1 μM. The co-operative effect was lost by point mutants of syntenin PDZ2 domain (K214A, K250A or N215D), although the mutants were still able to bind PIP_2_ (Supplementary Fig. 3a–c). Pre-incubating wild-type PDZ2 with mutant Frizzled 7 peptides (AGETAV or EGETAV) also failed to reveal co-operativity (Fig. 3e). The sensorgrams obtained with the PDZ2 K214A/K250A mutant clearly indicate that specific binding to liposomes-containing PIP_2_ is lost after double mutation and that pre-incubation with Frizzled 7 does not help PIP_2_ binding (Fig. 3c,d).

Previous studies have indicated a role for PDZ1 in Frizzled 7 and PIP_2_ binding^17^,^18^,^34^. To clarify to what extent a cooperative binding could be attributed to the PDZ1 domain, wild-type PDZ1 domain was perfused over composite liposomes. The responses observed when PDZ1 was perfused over composite liposomes were much higher than for PDZ2. A specific PIP_2_ interaction could be detected at high concentrations (80–180 μM) of protein (Supplementary Fig. 3d). Pre-incubation with 2 mM of KGETAV peptide had no effect on responses observed with PI-containing liposomes, but surprisingly decreased responses observed with PIP_2_-containing liposomes (Supplementary Fig. 3e).

Taken together, these biophysical experiments support a model where the PDZ2 domain of syntenin mediates co-operative Frizzled 7 and PIP_2_ binding.

### Frizzled 7-PIP_2_-syntenin PDZ1-PDZ2 co-operative binding

**Figure 4:**
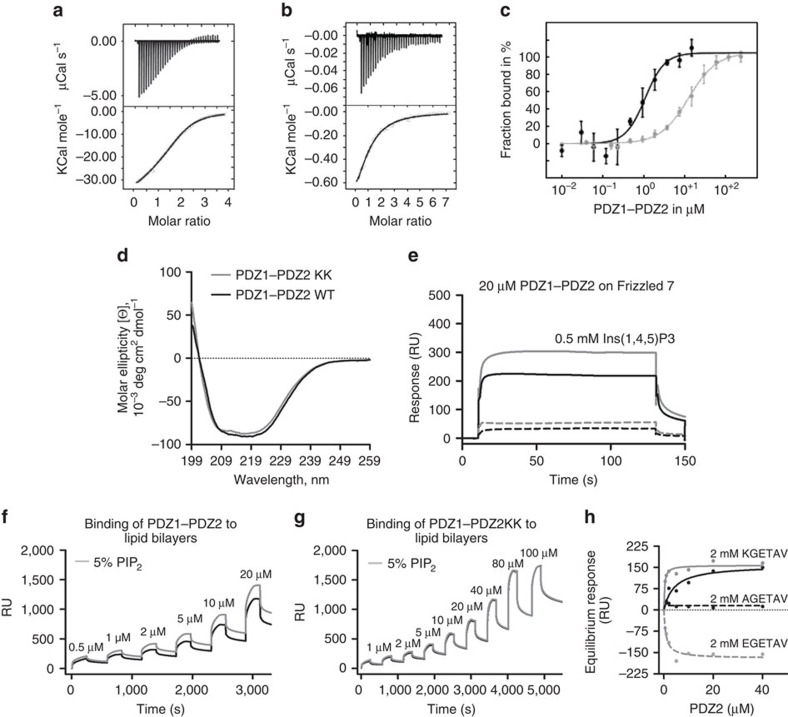
Frizzled 7 and PIP_2_ binding by the syntenin PDZ1–PDZ2 tandem. (**a**) Isothermal titration calorimetry (ITC) titration of the PDZ1–PDZ2 tandem with Frizzled 7 8mer peptide. (**b**) ITC titration of the PDZ1–PDZ2 tandem with Ins(1,4,5)P3. Top half graphs (**a**,**b**) show data after base line correction and bottom half graphs show the integrated data corrected for the heat of dilution of ligands. (**c**) Titration curve for the interaction of the PDZ1–PDZ2 tandem with Frizzled 7 peptide in absence (grey symbols) or presence of Ins(1,4,5)P3 head group (black symbols) as measured by microscale thermophoresis (MT). The solid curves represent the best fit of the Hill’s equation to the experimental data. Error bars represent s.d. of the mean. (**d**) Circular dichroism (CD) experiments illustrating similar structural properties for the PDZ1–PDZ2 tandem wild-type (black line) and mutant K214A/K250A (grey line). (**e**) Blank-subtracted SPR sensorgrams. Binding of PDZ1–PDZ2 tandem wild-type (black continuous line) or PDZ1–PDZ2 tandem mutant K214A/K250A (black dotted line; 20 μM) over immobilized Frizzled 7 19mer. A fixed concentration of 0.5 mM Ins(1,4,5)P3 (IP_3_, PIP_2_ head group) was pre-incubated with PDZ1–PDZ2 tandem wild-type (grey continuous line) or PDZ1–PDZ2 tandem mutant K214A/K250A (grey dotted line; 20 μM) before perfusion over immobilized Frizzled 7 19mer. (**f**,**g**) Sensorgrams illustrating the binding of increasing concentrations of the PDZ1–PDZ2 tandem wild-type (**f**) and the PDZ1–PDZ2 tandem mutant K214A/K250A (**g**) to liposomes containing 30% PC/40% PE/20% PS and either 10% PI or 5% PI and 5% PIP_2_. (**h**) The signals obtained at equilibrium on the 5% PIP_2_-containing liposomes for PDZ1–PDZ2 tandem in the absence (black continuous line) or in the presence of 2 mM of different 6mer Frizzled 7 peptide as indicated (KGETAV, grey continuous line; AGETAV, black dotted line; EGETAV, grey dotted line) were subtracted for association to 10% PI liposomes and plotted as a function of protein concentration. (**a**,**b**,**d**–**h**) The data show one experiment representative of at least three independent experiments done in duplicate. RU, response units. See Supplementary Tables 1 and 2 and Supplementary Fig. 4 for complementary information.

We further investigated the co-operative effect in the context of the full PDZ1–PDZ2 tandem of syntenin because this tandem of closely linked PDZ domains has been shown to form an integral structural and functional unit or ‘supramodule’^35^,^36^. First, we characterized the binding and stoichiometry of the interaction with a single ligand by isothermal titration calorimetry (ITC) experiments. We used the six residues C-terminal peptide from Frizzled 7 and IP_3_, the head group of PIP_2_ (Fig. 4a,b; Supplementary Table 1). As expected for Frizzled 7, the experimental data (Fig. 4a) fit best to a binary sequential binding model with dissociation constants of 20.5±0.4 μM for the first site and 4.3±0.2 μM for the second site. On the other hand, the binding isotherm of IP_3_ (Fig. 4b) is best described by a single binding site with a dissociation constant of 21.8±0.3 μM.

The analysis of the thermodynamic parameters (Supplementary Table 1) shows peptide binding is enthalpy driven and entropy unfavored. This correlates with the network of H-bonds that arise from the β-addition resulting from the binding to the PDZ domain and the concomitant reduction in degrees of freedom of the bound peptide. The binding of the head group of PIP_2_ is by contrast entropy driven and slightly enthalpy favourable. This is typically a signature of the binding of hydrophobic ligands driven by the increase entropy of water molecules released from the hydration layer of the head group of PIP_2_ upon binding. Similar binding results were obtained in equivalent experiments using microscale thermophoresis (MT), which also confirmed that the interaction between the PDZ1–PDZ2 tandem and Frizzled 7 is reinforced by the presence of IP_3_ (Fig. 4c; Supplementary Table 2). In these experiments, the dissociation constant for Frizzled 7 decreased one order of magnitude to 1.2±0.2 μM with a Hill’s coefficient of 1.8.

We further investigated the positive co-operativity of the syntenin wild-type PDZ1–PDZ2 tandem and its K214A/K250A mutant in a series of SPR experiments. The structural integrity of the mutant was confirmed by circular dichroism (CD; Fig. 4d). In these SPR experiments, PDZ1–PDZ2 binds to the 19 residues C-terminal Frizzled 7 peptide with an apparent *K*_D_ value of 8±1 μM. The dissociation constant was improved to 2±1 μM in the presence of 0.5 mM of IP_3_ (Fig. 4e; Supplementary Fig. 4a,e). Compared to wild-type syntenin PDZ tandem, the binding of the K214A/K250A mutant tandem was significantly reduced (Fig. 4e; Supplementary Fig. 4c) and the co-operative binding was lost (Fig. 4e; Supplementary Fig. 4d). As for PDZ2, the mutations affected Frizzled 7 binding and this reduced binding could not be compensated by pre-incubation with IP_3_ (Supplementary Fig. 4e).

We further tested the binding of the PDZ tandem to PIP_2_ using composite liposomes. The wild-type PDZ1–PDZ2 shows a clear specific PIP_2_ binding (Fig. 4f), in contrast to the PDZ1–PDZ2 mutant K214A/K250A (Fig. 4g). The interaction of wild-type PDZ1–PDZ2 with PIP_2_-containing liposomes was increased upon pre-incubation with wild-type Frizzled 7 peptide (KGETAV), but not with the mutant AGETAV or EGETAV peptides (Fig. 4h).

Taken together, these experiments indicate that the PDZ1–PDZ2 supramodule supports co-operative Frizzled 7 and PIP_2_ binding, as suggested by the structure.

### Tripartite interactions control Frizzled 7 cell localization

**Figure 5:**
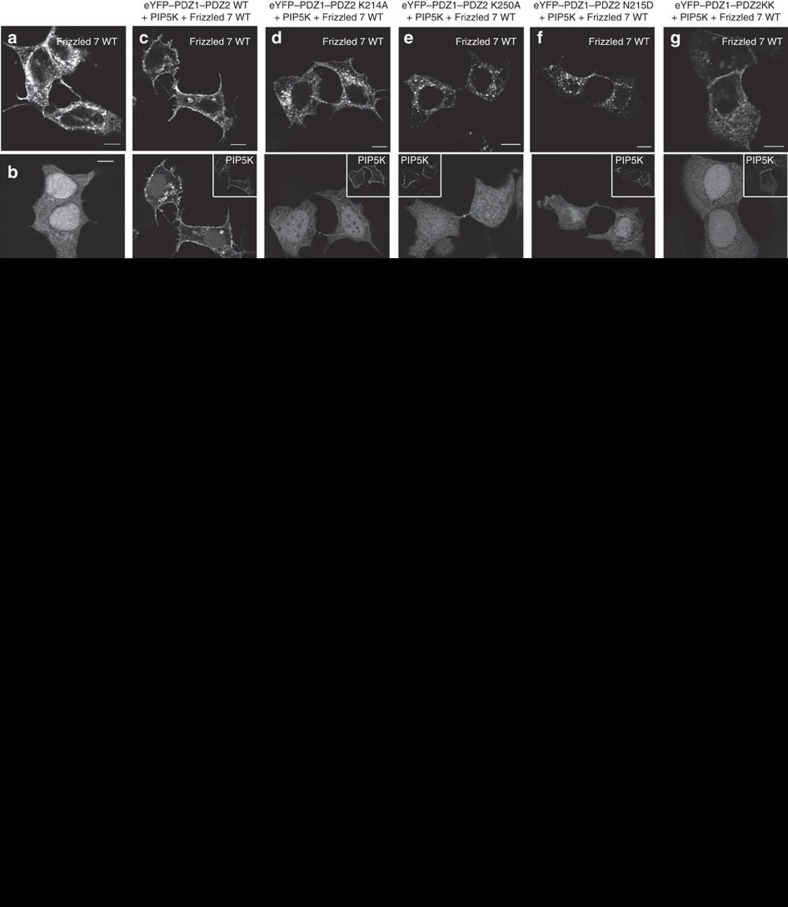
Residues in syntenin and Frizzled 7 important for tripartite interaction with PIP_2_ support Frizzled 7 trafficking to the plasma membrane. Representative confocal micrographs (**a**–**l**) of MCF-7 cells showing the distribution of Frizzled 7, eYFP–PDZ1–PDZ2 tandem wild type or mutants (K214A, K250A, N215D or K214A/K250A), phosphatidylinositol 4-phosphate 5-kinase (PIP5K), and mCherry-ARF6T27N as indicated. In **a**, cells express solely Frizzled 7 wild type, in **b**, cells express solely eYFP–PDZ1–PDZ2 tandem wild type. (**m**) Analysis of protein localization for each cell was performed by tracing a line intensity profile across the cell. *I*_mb_ is the fluorescence intensity at the plasma membrane and *I*_cyt_ is the average cytosolic fluorescence intensity. *R*_max_ is the percentage of protein in plasma membrane. (**n**) Bar graph showing the relative abundance at the plasma membrane of Frizzled 7 (white bars) when co-expressed with different molecules as indicated at the bottom. Values correspond to mean relative intensities±s.d. calculated for 20 cells in at least three independent experiments. Scale bar, 10 μm. Note that the plasma membrane enrichment of Frizzled 7 as seen when co-expressed with PIP5K and eYFP–PDZ1–PDZ2 tandem is lost upon mutations of syntenin (K214 to A, K250 to A or N215 to D) and Frizzled 7 (K-5 to A or E) or upon expression of mCherry-ARF6T27N (blocking recycling from endosomes to the plasma membrane). The plasma membrane enrichment of the eYFP–syntenin PDZ tandem constructs is similarly affected. The statistical significance was performed by one-way analysis of variance followed by Tukey’s *post hoc* test. *P*<0.05 was considered as significant. Statistical tests were performed using GraphPad Prism 5 software. **P<0.01, ***P<0.001. See Supplementary Fig. 5 for more representative micrographs. Supplementary Fig. 6 illustrates that mutations affecting the coincident detection of Frizzled 7 and PIP_2_ by syntenin PDZ2 domain result in the localization of the receptor in recycling endosomes, as does the expression of the ARF6 dominant negative for recycling (ARF6T27N).

To test the biological significance of the co-operativity of peptide and PIP_2_ binding, we performed microscopy experiments with MCF-7 cells transiently transfected with expression vectors for eYFP-tagged PDZ1–PDZ2, non-tagged Frizzled 7 full-length receptor, and/or myc-tagged phosphatidylinositol 4-phosphate 5-kinase (PIP5K) that increases the cellular PIP_2_ levels^37^,^38^. When overexpressed alone in freshly plated MCF-7 cells, wild-type Frizzled 7 receptor (Fig. 5a; Supplementary Fig. 5a,m), and its mutant K-5A (Fig. 5h; Supplementary Fig. 5h,t) and K-5E (Fig. 5i; Supplementary Fig. 5i,u) localize poorly at the plasma membrane and are rather found inside the cell but outside the nucleus. Similarly, overexpressed eYFP–PDZ1–PDZ2 is not enriched at the plasma membrane (Fig. 5b,f; Supplementary Fig. 5b,n). However, when co-expressed, Frizzled 7 and eYFP–PDZ1–PDZ2 strongly localize to the plasma membrane, an effect that is further exacerbated by PIP5K co-expression (Fig. 5c,n; Supplementary Fig. 5c,o). This translocation is lost when eYFP–PDZ1–PDZ2 is replaced by the PDZ1–PDZ2 mutant K214A (Fig. 5d,n; Supplementary Fig. 5d,p); K250A (Fig. 5e,n; Supplementary Fig. 5e,q); N215D (Fig. 5f,n; Supplementary Fig. 5f,r) or K214A/K250A (Fig. 5g,n; Supplementary Fig. 5g,s) or when Frizzled 7 is replaced by the Frizzled 7 mutant K-5A (Fig. 5j,n; Supplementary Fig. 5j,v) or K-5E (Fig. 5k,n; Supplementary Fig. 5k,w). These data indicate that co-operative binding with PIP_2_ is required for translocation to the plasma membrane. We then characterized the subcellular localization of Frizzled 7 in the conditions where it failed to reach the plasma membrane by confocal microscopy and found that it was co-accumulating with the transferrin receptor in recycling endosomes (Supplementary Fig. 6). This is reminiscent of and consistent with a previous study, showing that syntenin can stimulate the recycling of syndecan to the plasma membrane when it interacts with PIP_2_^19^. This effect of syntenin was shown to rely on the activation of the small GTPase ADP ribosylation factor 6 (ARF6). This GTPase recruits PIP5K to the recycling endosomes and stimulates plasma membrane targeting of syndecan provided with syntenin able to interact with PIP_2_. To assess whether Frizzled 7 plasma membrane translocation similarly relies on ARF6 recycling, we evaluated the Frizzled 7 distribution in cells co-expressing eYFP–PDZ1–PDZ2 wild type, PIP5K and mCherry-ARF6T27N, a dominant negative form of ARF6 blocking ARF6-dependent recycling^39^. As expected, the plasma membrane distribution of Frizzled 7 was then strongly decreased and similar effects were observed on eYFP–PDZ1–PDZ2 (Fig. 5l,n; Supplementary Fig. 5l,x). Taken together, these results highlight the pivotal role of PIP_2_ binding by the PDZ2 domain of syntenin in the membrane trafficking of its peptide cargo.

### Tripartite interactions support c-jun phosphorylation

**Figure 6:**
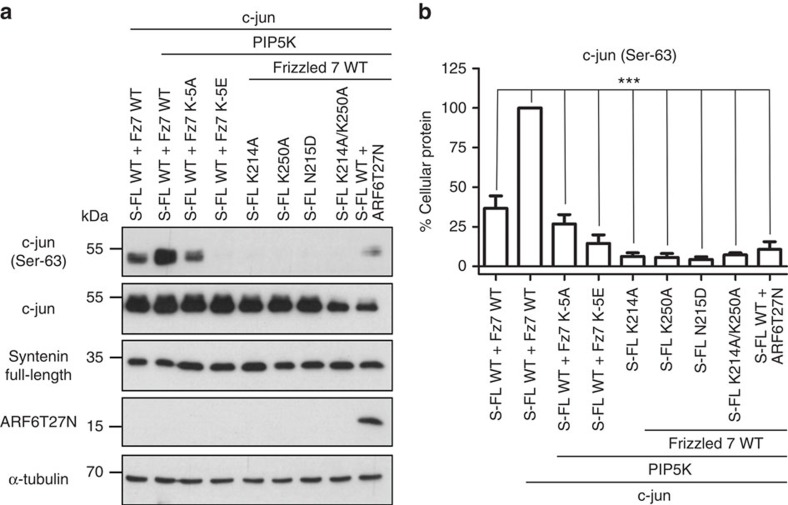
Frizzled 7-PIP_2_-syntenin coincident detection is required for plasma membrane localization and c-jun phosphorylation. (**a**) Cell lysates originating from HEK293T cells transiently overexpressing proteins as mentioned on top of the gel, were tested in western blotting for c-jun phosphorylation (Ser-63). α-tubulin was used as a loading control and the respective expression of syntenin full-length (S-FL; antibody detecting non-tagged wild-type or mutants) and total c-jun are also shown. Note the marked effect of the various mutations and the ARFT27N (dominant negative mutant for recycling, last lane) on c-jun phosphorylation. (**b**) Bar graph showing the relative percentage of cellular c-jun phosphorylation (c-jun Ser-63; white bars) when co-expressed with different molecules as indicated below. The statistical significance was performed by one-way analysis of variance followed by Tukey’s *post hoc* test. *P*<0.05 was considered as significant. Statistical tests were performed using GraphPad Prism 5 software. **P<0.01, ***P<0.001.

The potential role of syntenin–Frizzled 7–PIP_2_ interaction in Wnt signalling was further investigated by measuring the phosphorylation of the c-Jun-NH_2_-terminal kinase (JNK) substrate c-jun, an established downstream target of non-canonical Wnt signalling^40^,^41^. For these signalling experiments, we used HEK293T cells and non-tagged full-length syntenin and Frizzled 7 constructs. Co-expression of wild-type syntenin and Frizzled 7 led to c-jun phosphorylation, which was increased when PIP5K was co-expressed (Fig. 6a, lanes 1–2; Fig. 6b). We then investigated whether stimulation of c-jun phosphorylation would be affected by the expression of the mutants that do not support Frizzled 7–PIP_2_–syntenin plasma membrane localization. For none of them we observed levels of c-jun phosphorylation equivalent to those observed for wild-type proteins (Fig. 6a, compare lane 2 with lanes 3–8; Fig. 6b). Finally, blocking ARF6 recycling by co-overexpressing the ARF6T27N mutants with wild-type syntenin, Frizzled 7 and PIP5K also impaired c-jun phosphorylation (Fig. 6a, lane 9; Fig. 6b). Taken together, these results highlight the pivotal role of PIP_2_ binding by the PDZ2 domain of syntenin in the signalling of its Frizzled 7-peptide cargo.

## Discussion

In an attempt to better understand how syntenin PDZ tandems integrate peptide and lipid binding, we determined the crystal structure of the syntenin PDZ tandem in complex with a C-terminal hexapeptide from Frizzled 7 and the lipid PIP_2_. We used a PDZ tandem slightly longer at the N terminus and the C terminus (amino acids 107–275) than other crystallization studies (amino acids 113–273 (refs 42, 43)) because our binding and cell biology data suggest that these optimized domain boundaries better preserve the function of the PDZ tandem supramodule^10^,^11^. Our ITC data, as well as previous work^17^, point towards two binding sites for Frizzled 7 in the syntenin PDZ tandem. In our crystal structure, Frizzled 7 peptide binds only to PDZ2. PDZ1 adopts a conformation expected for an empty PDZ domain^43^, and this conformation is stabilized by lattice contacts. More surprising is the absence of bound PIP_2_ to the PDZ1 domain. As in the case of Frizzled 7, it has been documented that, in isolation, both PDZ domains are able to bind PIP_2_ and that the PDZ tandem outperforms the single PDZ domains^18^.The present study corroborates these conclusions. Yet, using glutathione S-transferase (GST)-tagged proteins (known to form dimers via their GST moiety, but also to help stability) our study from 2002 concluded that when PIP_2_ is embedded in liposomes mimicking the phospholipid composition of the plasma membrane (as in this study), PDZ1 outperforms PDZ2. Here we used his-tagged proteins to avoid GST-dimerization-based effects (also after ensuring that the 6-His-tag does not influence the lipid measurements). With these constructs, PDZ2 outperforms PDZ1 for the selective recognition of composite liposome-embedded PIP_2_. In addition, the PDZ tandem appears to lose its ability to selectively recognize PIP_2_ when the PDZ2 is mutated for PIP_2_ interaction. Moreover our ITC and MT data suggest only one specific PIP_2_-binding site in the PDZ tandem. A plausible explanation, which would conciliate the past and present observations, would be that PDZ1 significantly contributes to the membrane recognition, but poorly contributes to the selective PIP_2_ recognition (unless fused to a GST tag) when compared with PDZ2, particularly if the latter is peptide loaded. The co-operative effect is not observed for PDZ1, nor with the domain in isolation, neither with a tandem mutated for the PDZ2 co-operative effect. In the tandem, PDZ1 might thus be primarily necessary to bring sufficient membrane phospholipid affinity. This would be fully consistent with the observation that the tandem is required for the membrane localization in cells, while single PDZ domains fail to do so^11^. Thus, a dual mode of membrane interactions in which the specific PIP_2_ binding present in the PDZ2 domain, supported by membrane interactions due to the PDZ1 domain, would support syntenin recruitment to the membrane. The cluster of positive residues located over the peptide-binding groove of the PDZ1 domain is likely contributing to electrostatic interactions with the membrane. This dual mode of membrane interaction using both electrostatic recruitment and specific phosphoinositide binding is common in membrane-binding proteins^44^ and is present in PDZ domain proteins^7^. In particular, specific PIP_2_ binding and general electrostatic recruitment has been reported for the PDZ domain of the *Drosophila* protein polychaetoid that binds PIP_2_ in cells^22^. Mutations in PDZ1 were shown to abolish syntenin PDZ tandem binding to PIP_2_ in cells^18^,^19^, but in the light of the present study, a fine-tuned interpretation would be that these mutations preclude electrostatic interactions necessary, but not sufficient for the recognition of cellular PIP_2_. The PIP_2_-binding site found in the PDZ2 domain shows neither steric hindrances nor electrostatic incompatibilities. In fact, the PIP_2_ head group fits properly into the binding site, with the backbone of Frizzled 7 and the side chain of residues K214, N215 and K250 from the PDZ2 domain oriented towards it, seemingly clipping the head group of the PIP_2_ in place. The configuration of the site is similar to those observed in other PIP_2_-binding proteins, with the same residues, lysine and asparagine, and within the same distance range. The tight network of interactions created upon binding of the PIP_2_ molecule, but more importantly the presence of D251 and the lack of space to fit any another phosphate group in the third position of the head group in the lipid-binding site, explains the high selectivity towards PIP_2_ (ref. 18).

Interestingly, Sugi *et al*.^45^ proposed a model for PIP_2_ binding of the PDZ2 domain of syntenin based on the phosphate-bound structure of the PDZ domain of the non-homologus protein tamalin. The model remarkably predicts the role of lysines 214 and 250 on PIP_2_ binding. However, other residues previously anticipated to be involved in PIP_2_ binding are not observed in our crystal structure. In particular, the residues of the carboxylate-binding loop predicted to participate in lipid binding only take part in canonical peptide binding. The model from Sugi predicts that the aliphatic tails of the lipid would displace peptides bound in the canonical mode, as one aliphatic tail with only eight carbons was modelled into the peptide-binding groove. This implies that the binding would provide enough energy to remove one aliphatic tail from the membrane core, and leave 10–12 aliphatic carbons free outside the peptide-binding groove. Instead our model shows how peptide and lipid head group can simultaneously interact with the aliphatic tails of the lipid probably still inside the membrane core.

Noteworthy, peptide and PIP_2_ were so far thought to cooperate to bind syntenin PDZ tandem, but to compete for single domain binding^18^. In retrospect, data supporting the competition could be specific to particular experimental settings. Indeed, these data were obtained from pull-down experiments, where peptides were coated on beads, and syntenin PDZ domains and PIP_2_ were in solution. Yet, competition was observed for concentrations of PIP_2_ above the critical micellar concentration. The two PDZ ligands were thus present in different ‘phases’, possibly explaining the lack of simultaneous binding and *a fortiori* the failure to observe co-operative binding. The co-operative binding of the tandem was confirmed in the present study. Yet, unexpectedly we found that a single PDZ domain can also support co-operative binding between peptide and PIP_2_. Clearly, Frizzled 7 and PIP_2_ can engage into tripartite interactions with the second PDZ domain of syntenin. This co-operative binding of the PDZ2 in isolation was univocally demonstrated by the combination of our X-ray structure, the SPR data and the mutational analysis. Moreover, the data support a model where the PDZ2 is on its own responsible for the co-operativity of the tandem. Indeed, the co-operative binding of the tandem is lost when the PDZ2 domain is mutated. This study also established that tripartite PDZ–Frizzled 7–PIP_2_ interactions, although still very dynamic, can be one order of magnitude stronger than bipartite interactions.

The close proximity of the PIP_2_-binding site to the peptide-binding groove is a remarkable feature. The backbone of Frizzled 7 provides direct polar and van der Waals contacts to the PIP_2_-binding interface that stabilize the head group. This suggests that both the protein and the peptide form the full binding site for PIP_2_, and therefore the peptide reinforces the interaction with the lipid. Indeed, SPR experiments suggest that the addition of Frizzled 7 peptide potentiates more PIP_2_ interaction than PIP_2_ potentiates Frizzled 7 interaction. As PIP_2_ segregates in specific membrane compartments upon the activation/recruitment of lipid-modifying enzymes and as tripartite interactions display higher affinity than bipartite interactions, one might speculate that these properties help protein complexes to translocate from one subcellular compartment to another. Indeed, our cell biology experiments are indicative of such phenomena. When syntenin and Frizzled 7 are co-expressed together with PIP5K, their localization is shifted from recycling endosomes to the plasma membrane. This effect is lost with the syntenin PDZ2 mutants and also with Frizzled 7 mutants or with a mutant ARF6 small GTPase expected to interfere with PIP5K–PIP_2_ localized production. Such mutations also impair Frizzled 7 signalling such as c-jun phosphorylation that is an established readout of planar cell polarity activity and related activation of JNK signalling^46^,^47^.

In conclusion, this study provides X-ray structure data on PDZ–lipid interaction and sheds light on the molecular basis of a longstanding established functional crosstalk between syntenin PDZ domains, cognate peptides and membrane phosphoinositides in subcellular trafficking and signalling. In particular, it demonstrates that the PDZ2 domain of syntenin PDZ tandem can mediate coincident and most probably consecutive recognition of Frizzled 7 cytoplasmic domain and PIP_2_, and that this co-operative interaction supports the trafficking of Frizzled 7 to the plasma membrane in an ARF6/PIP_2_-dependent manner, as well as Frizzled 7 signalling. By highlighting key residues for specific PIP_2_ interaction in syntenin PDZ tandem, that were confirmed to be functionally important by mutational analysis in various readouts *in vitro* and in cells, it contributes to our molecular understanding of PDZ proteins as key molecules in membrane compartmentalization and dynamics with important impact for signalling, and paves the way for rational pharmacological interventions.

## Methods

### Lipids used in this study

For crystallization studies, we used L-α-phosphatidyl-(1,2-dibutanoyl)-D-myo-inositol 4,5-bisphosphate (C4-PIP_2_, formula C_17_H_33_O_19_P_3_) synthesized by SiChem (Germany). For experiments with liposomes, we used dipalmitoyl PIP_2_ (P-4516) from Echelon Biosciences. D-myo-Inositol 1,4,5-trisphosphate or IP_3_ (Q-0145), and biotin PIP_2_ or C6-PtdIns(4,5)P_2_ (C-45B6) were also purchased from Echelon Biosciences. 1-palmitoyl-2-oleoyl-sn-glycero-3-phosphocholine (PC-850457P); 1-palmitoyl-2-oleoyl-sn-glycero-3-phospho-L-serine (PS-840034P) and 1-palmitoyl-2-oleoyl-sn-glycero-3-phosphoethanolamine (PE-850757P) were obtained from Avanti Polar Lipids (Alabaster, Alabama, USA).

### Structure determination

The PDZ tandem was dialyzed overnight at 4 °C in pre-crystallization buffer (5.0 mM Tris pH 7.5, 150.0 mM NaCl, 1.0 mM EDTA and 1.0 mM DTT). The next day the PDZ tandem was concentrated, up to the concentration indicated in each figure legend, by ultra-filtration using Vivaspin 15, 2 and 500 centrifugal devices of 5 kDa molecular weight cut-off (MWCO) (Sartorius, Goettingen, Germany). The concentrated PDZ tandem was centrifuged at 17,000 *g* for 10 min at 4 °C on a bench top centrifuge to remove aggregated material, and then incubated at room temperature (25 °C) for 5 min with C4-PIP_2_ and the Frizzled 7 peptide (sixmer) in a molar ratio protein:lipid:peptide of 10:23:23. Crystallization conditions were screened using the Hampton Research Crystal Screen and the Crystal Screen 2 crystallization kits (Hampton Research, CA, USA). Drops of 1.0 μl of the protein:lipid:peptide stock solution at 13 mg ml^−1^ of protein were mixed with 1.0 μl drops of precipitant solution on siliconized glass cover slides and equilibrated over 500 μl of precipitant. The plates were incubated at 19 °C. Initially crystals were obtained condition 9 of Crystal Screen and these were optimized lowering the protein concentration up to 8.0 mg ml^−1^.

Before X-ray diffraction experiments, crystals were transferred to a cryoprotectant solution consisting of 0.2 M ammonium acetate, 0.1 M sodium citrate tribasic dihydrate pH 5.6 and 32.5% (w/v) polyethylene glycol (PEG) 4.000 and containing both peptide and lipid at a concentration of 0.965 mM. The crystals were vitrified directly in the nitrogen stream (100 °K). Data were collected on beamline X13 of the DESY synchrotron (Hamburg, Germany). The structure was solved by molecular replacement using the coordinates of the free PDZ domain of human syntenin (PDB entry: 1N99 (ref. 43) as search model. The coordinates from the head group of C4-PIP_2_ were docked into the map from the initial molecular replacement model resulting from PHASER^48^, and the Frizzled 7 peptide was also built from the same map. The resulting model was subsequently refined. The final model was obtained after alternating cycles of refinement with BUSTER^49^ and manual build using Coot^50^, and has an *R* of 18.3% and *R*_free_ of 23.9% (see Table 1 for the data collection and refinement statistics).

### Molecular biology

The complementary DNA of syntenin PDZ2 (coding for amino acids 196–275) and PDZ1–PDZ2 tandem (coding for amino acids 107–275) were sub-cloned into the eYFP-C1 (Invitrogen) for microscopy assays or into the pETM-11 vector (EMBL Heidelberg) for the expression of N-terminally His-tagged proteins from ER2566 cells^22^. Site-directed mutagenesis was performed using QuikChange protocol (Stratagene). The sequence of the coding region was confirmed by DNA sequencing. The Frizzled 7 and the PIP5K eukaryotic expression constructs were respectively non-tagged and myc-tagged.

### Cells experiments and microscopy

All cell lines were obtained from the American Type Culture Collection (Manassas, VA) and were routinely grown in DMEM/F12 medium (Life Technologies) supplemented with 10% fetal bovine serum (Gibco). They were controlled for the absence of mycoplasma contamination. For microscopic analysis, MCF-7 cells were plated on a 24-well plate, transfected using the FUGENE transfection reagent (Roche), fixed with 4% paraformaldehyde for 20 min, washed in PBS and then incubated with 10 μg/ml anti Frizzled 7 antibody (AF198, R&D systems), 5 μg/ml anti myc antibody (sc-40 AC, Santa Cruz), 10 μg/ml anti transferrin receptor (13-6800, Life Technologies) and appropriated Alexa-conjugated secondary antibodies (Molecular Probes) with PBS containing 1% BSA and 0.5% Tween 20. Coverslips were mounted on Mowiol-DABCO and the enrichment of eYFP–syntenin PDZ tandem at the plasma membrane was scored with a Zeiss Meta confocal microscope (LSM 510 META, Zeiss, France) with a UV laser and × 63 objective. Confocal images were analysed using ImageJ and Photoshop (Adobe, San Jose, CA) software. An individual analysis of protein localization for each cell was performed by tracing a line intensity profile across the cell as described^51^. The relative increase in the plasma membrane localization of the protein was calculated by using the ratio:


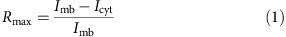


where *R*_max_ is the percentage of protein at the plasma membrane, *I*_mb_ is the fluorescence intensity at the plasma membrane and *I*_cyt_ is the average cytosolic fluorescence intensity. For c-jun phosphorylation assays, HEK293T cell extracts were prepared, separated on 10% SDS–polyacrylamide gel electrophoresis and transferred onto a nitrocellulose membrane. The BSA-blocked membranes were incubated with 1/1,000 anti phospho c-jun (Ser-63, 2361, clon54B3, Cell Signaling Technologies), 1/1,000 anti c-jun (9165, clon 60A8, Cell Signaling Technologies), 1/10,000 anti α-tubulin (T6199, Sigma-Aldrich), 1/500 anti ARF6 (sc-7971, Santa Cruz) and home-made anti-syntenin antibodies characterized previously^52^. Signals were visualized with enhanced chemiluminescence detection reagent (Amersham Pharmacia Biotech) and were quantified by densitometric scanning using ImageJ. Full scans of the western blots are supplied in Supplementary Fig. 7.

### Size-exclusion chromatography

Analytical size-exclusion chromatography was performed using an Akta Explorer system and a Superdex 75 10/300 GL column (GE Healthcare). The column was equilibrated in 25 mM HEPES, 150 mM NaCl, pH 7.4 buffer and a set of molecular weight standards were used for the calibration (conalbumine, ovalbumine, carbonic anhydrase, ribonuclease and aprotinin; GE Healthcare).

### Preparation of vesicles

Lipid vesicles were generated by mixing chloroform solutions of 1-palmitoyl-2-oleoyl-*sn*-glycero-3-phosphocholine, 1-palmitoyl-2-oleoyl-*sn*-glycero-3-phospho-L-serine, 1-palmitoyl-2-oleoyl-*sn*-glycero-3-phosphoethanolamine, dipalmitoyl-PI and dipalmitoyl-PIP_2_ in the desired proportions. Lipids were dried under a stream of nitrogen followed by exposure to high vacuum for 30 min. Dried phospholipids were resuspended in the corresponding buffer (25 mM HEPES, pH 7.4 and 150 mM NaCl) by vigorous vortexing and also using the bath sonicator to get all of the lipid into suspension. Then the lipid suspension was subjected to eight rounds of freeze (liquid N2)-thaw in the bath sonicator at 40 °C. The large unilamellar phospholipid vesicles of ∼100 nm diameter were prepared by extruding rehydrated phospholipid suspensions through two stacked 0.1 μm polycarbonate membranes.

### Surface plasmon resonance experiments

All SPR measurements were carried out at 25 °C using a BIAcore T200 instrument (GE Healthcare). A total of 100 resonance units (RU) of biotinylated ligands corresponding to the 19 amino acids (SWRRFYHRLSHSSKGETAV) that compose part of the cytoplasmic domain of Frizzled 7 wild-type or Frizzled 7 K-5A mutant (SWRRFYHRLSHSSAGETAV; GenScript), and the biotinylated C6-PtdIns(4,5)P_2_ were immobilized on a streptavidin-sensor chip (GE Healthcare). Analytes (His-tag fusion proteins) were perfused at 30 μl per min in running buffer (10 mM HEPES pH 7.4, 150 mM NaCl and 0.005% Tween 20) at different concentrations. In this case, the reference channel was blank immobilized. The injection time was 120 s, long enough for the sensorgrams to reach equilibrium, and the dissociation time was 90 s. The surfaces were regenerated between runs by short pulses of 50 mM NaOH and 1 M NaCl at 30 μl per min flow rate. Sensorgrams were corrected for binding to reference surfaces and for buffer effects (blank subtracted) before further data analysis. Equilibrium dissociation constants (*K*_D_) were calculated by fitting the data to a simple Langmuir binding isotherm by using GraphPad Prism. For determination of apparent *K*_D_ values the response in RU at equilibrium was plotted as function of protein concentration. The experiments were repeated at least three times using different preparations of proteins and streptavidin-sensor chips. *K*_D_ values are given plus–minus s.d.’s.

### Liposome-binding experiments

The L1 chip (GE Healthcare) was coated with composite liposomes (30% PC/40% PE/20% PS/5% PI and 5% PIP_2_ or 10% PI for background reference). Liposomes were injected at a flow rate of 5 μl per min until total immobilization reached 5,000 RU. A short pulse of 10 mM NaOH at 100 μl per min was performed to remove non-attached liposomes before measurements. Purified proteins were diluted in running buffer (25 mM HEPES, pH 7.4 and 150 mM NaCl) to the concentrations indicated and perfused at a flow rate of 30 μl per min. To carry out these SPR experiments, we employed the method known as single-cycle kinetics described in 2006 by Karlsson and co-workers. It consists in injecting the analyte at increasing concentrations, without regeneration steps between each sample injection to avoid that liposomes can be damaged after regeneration steps^53^. Previously to the binding experiments, we confirmed that the 6mer Frizzled 7 peptide did not interact with the lipid surface.

### Isothermal titration calorimetry experiments

For ITC experiments, the protein samples were extensively dialyzed at 4 °C against ITC buffer (10 mM Mes pH 7.0 and 5 mM β-mercapthoethanol). Protein concentrations were adjusted with the same buffer from 30 μM to 60 μM. Both ligands, the 8mer C-terminal peptide from Frizzled 7 (JPT, Germany) and IP_3_ (Sichem, Germany) were chemically synthesized. The ligands were dialyzed or dissolve on the same buffer at final concentration of 1.0 mM (Frizzled 7) or 2.0 mM (IP_3_). All samples were degassed without stirring and loaded on a VP-ITC microcalorimeter (MicroCal) and 32 injections of 8 μl were performed for each ligand. Origin software was used for data integration and fitting to the one site or two sites binding models by Marquardt methods as provided in the MicroCal Origin routines. *K*_D_ values are given±s.e.m.

### Microscale thermophoresis experiments

For MT experiments 6mer C-terminal peptides from Frizzled 7 and syndecan2, where chemically synthesized and N-terminally labelled with carboxifluorescein (JPT, Germany). The experiments were carried out in the same buffer as ITC experiments. Proteins and peptides were dialyzed or dissolve in this buffer. The peptide concentration was adjusted in the range from 40 to 320 nM. For a fix peptide concentration, the protein was serially diluted in the range of 40 nM 120 uM, mixed with peptide and let it equilibrate for 5 min at room temperature before loading into hydrophilic capillaries (NanoTemper, Germany) measuring on a Monolight NT.115 instrument equipped with red and green light-emitting diodes (also from NanoTemper). For each concentration point 5 s of cold fluorescence (base line) follow by 30 s of laser heating (at power indicated on each figure legend) and 5 s of cooling were registered. The data was analysed with NanoAnalyze software (NanoTemper) and fitted to the following binding equation derive from the law of mass action:


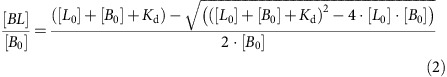


Where 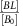 is the fraction of sites occupied, [*L*_0_] is the amount of added protein to each data point, [*B*_0_] is the total peptide concentration and *K*_d_ is the dissociation constant of the binding reaction.

For experiments where the affinity towards the head group of PIP_2_ was measured, the protein was fluorescently labelled with Monolith NT protein labelling kit RED (NanoTemper) according to the manufacturer’s indications. After labelling the protein was dialyzed in ITC buffer and then adjusted to a fix concentration in the range of 40–320 nM. IP_3_ was dissolve in the same buffer and serially diluted in the range of 40–1.0 mM. The same mixing, loading, data recording and data analysis parameters as in the case of peptide binding were applied. *K*_D_ values are given±s.e.m.

### Circular dichroism experiments

Purified PDZ2 and PDZ1–PDZ2 wild-type proteins and their respective mutant K214A/K250A were dialyzed extensively at 4 °C against buffer containing 25 mM sodium phosphate pH 7.5 and 50 mM NaCl. CD spectra were measured on a Jasco 815 CD spectrometer with a 2 mm pathlength cuvette. All CD spectra is average of three measurements taken at 25 °C.

### Dynamic light scattering experiments

Dynamic light scattering (DLS) measurements were performed on a Wyatt DynaPro NanoStar at a laser wavelength of 660 nm. Purified PDZ2 wild-type and mutant K214A/K250A were diluted in HEPES buffer (25 mM HEPES, 150 mM NaCl, pH 7.4) to a final concentration of 0.25 mg ml^−1^. Data were collected at 25 °C with an acquisition time of 5 s and the diameter size was averaged over 3 × 10 runs.

### Size-exclusion chromatography coupled with Multi Angle Light Scattering

SEC–multi angle light scattering (MALS) was performed using a Shimadzu Prominence HPLC (Kyoto, Japan) equipped with a Shodex KW402.5-4F column (Showa Denko Europe GmbH, Germany) connected to a Dawn Heleos II MALS detector equipped with a 660-nm laser and in-line T-rEX refractive index detector (Wyatt Technology Inc., California, USA). An aliquot of the protein was loaded onto the column and eluted at a flow rate of 0.2 ml per min in PBS buffer. The molar mass of pure protein was calculated from the observed light scattering intensity using a refractive index increment (dn/dc) of 0.185 ml g^−1^ as implemented in the ASTRA software (Wyatt Technology Inc.).

### Data availability

The data that support the findings of this study are available from the authors on reasonable request, see author contributions for specific data sets.

## Additional information

**How to cite this article:** Egea-Jimenez, A. L. *et al*. Frizzled 7 and PIP_2_ binding by syntenin PDZ2 domain supports Frizzled 7 trafficking and signalling. *Nat. Commun.* 7:12101 doi: 10.1038/ncomms12101 (2016).

## Supplementary Material

Supplementary Information Supplementary Figures 1-7 and Supplementary Tables 1-2

## Figures and Tables

**Table 1:** Data collection and refinement statistics.

	PDZ1–PDZ2 tandem·Fz7(6mer)·C4-PIP2
*Data collection*	
Space group	P 4_1_2_1_2
Cell dimensions	
*a*, *b*, *c* (Å)	71.74, 71.74, 126.36
α,β,γ (°)	90.00, 90.00, 90.00
Resolution (Å)	20.0–2.45 (2.54–2.45)
*R*_sym_ or *R*_merge_	0.18 (0.65)
*I* / σ*I*	15.3 (4.23)
Completeness (%)	99.6 (99.5)
Redundancy	12.4 (13.1)
	
*Refinement*	
Resolution (Å)	2.45
No. of reflections	175,324
No. unique reflections	12,233
*R*_work_/*R*_free_	19.7/25.4
No. atoms	2,744
Macromolecules	2,537
Ligands	48
Water	159
B factors (Å^2^)	38.2
Wilson’s plot	39.1
Protein	
Ligands	50.3
Peptide	59.6
C4-PIP2	35.1
Water	
Ramachandran profile	
Core (%)	92.8
Allowed regions (%)	6.3
Outliers (%)	0.9
r.m.s.d.	
Bond lengths (Å)	0.014
Bond angles (°)	1.39

*Deposition*	
PDB entry	4Z33

No., number, PDB, protein data bank; r.m.s.d., root mean squared deviation.
