# Supplementary material for: Frizzled 7 and PIP2 binding by syntenin PDZ2 domain supports Frizzled 7 trafficking and signalling
Source: Nat Commun. 2016 Jul 8;7:12101. doi: 10.1038/ncomms12101 (PMC5515355; doi:10.1038/ncomms12101)
Supplement: Supplementary Information — Supplementary Figures 1-7 and Supplementary Tables 1-2 [file ncomms12101-s1.pdf]

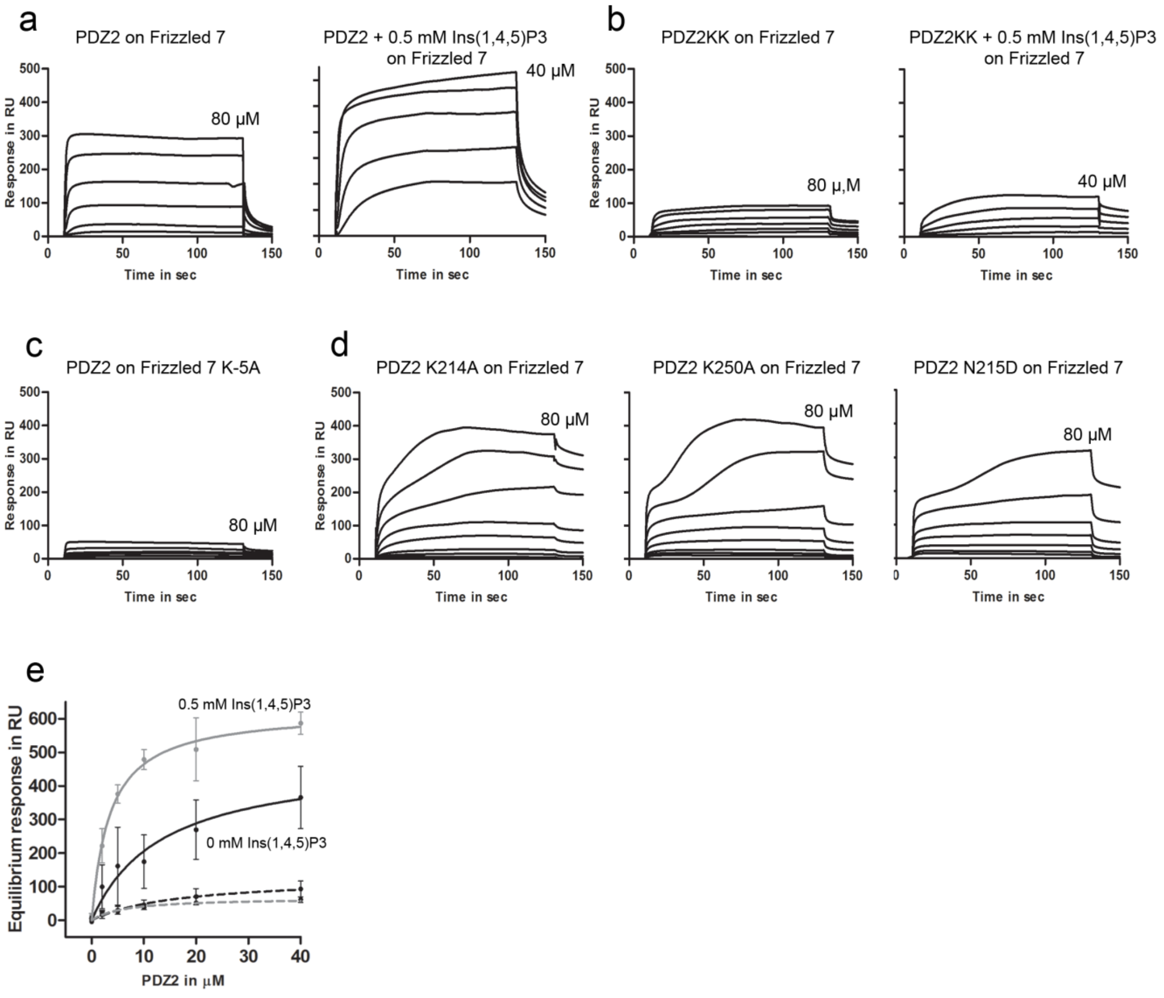

Supplementary Figure 1. SPR analysis of syntenin PDZ2-Frizzled 7 interaction. Blank reference-substracted SPR sensorgrams obtained for the binding of PDZ2 wild-type (**a**-left panel) or PDZ2 mutant K214A/K250A (**b**-left panel) (concentrations ranging from 2 to 80 µM) to immobilized Frizzled 7 wild-type 19mer. To test the formation of the tripartite complexes, we perfused PDZ2 wild-type (**a**-right panel) or PDZ2 mutant K214A/K250A (**b**-right panel) in the presence of 0.5 mM of Ins(1,4,5)P3 over immobilized Frizzled 7 wild-type. **c**. Blank reference-substracted SPR sensorgrams obtained for the binding of PDZ2 wild-type (concentrations ranging from 2 to 80 µM) to immobilized Frizzled 7 K-5A. **d**. Blank reference-substracted SPR sensorgrams obtained for the binding of PDZ2 single mutants as indicated on top (K214A, K250A or N215D) (concentrations ranging from 2 to 80 µM) to immobilized Frizzled 7 wild-type 19mer. **e**. Equilibrium binding isotherms of PDZ2 wild-type (black continuous line) or PDZ2 mutant K214A/K250A (black dotted line) in the absence or in the presence of 0.5 mM of Ins(1,4,5)P3 (grey continuous line for PDZ2 wild-type and grey dotted line for PDZ2 mutant K214A/K250A). Sensorgrams are representative of three independent experiments. Bars refer to ± s.d.

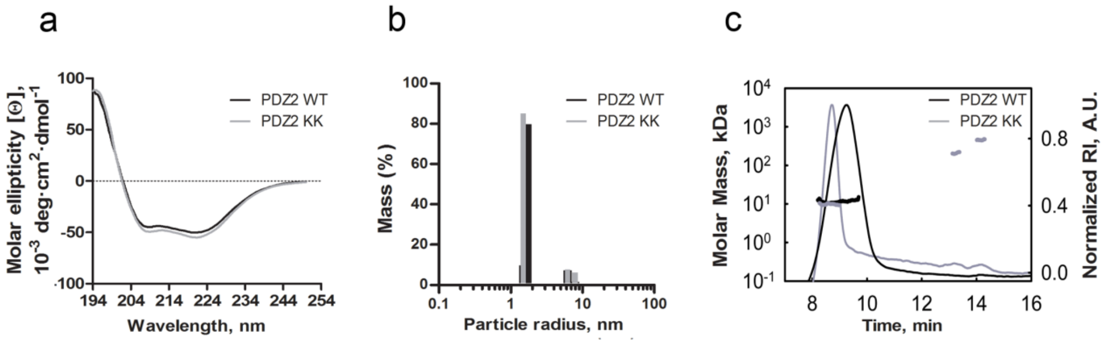

Supplementary Figure 2. PDZ2 wild-type and mutant K214A/K250A present a similar secondary structure and a soluble state. **a**. Circular dichroism (CD) spectra illustrating similar structural properties for the PDZ2 wild-type (black line) and mutant K214A/K250A (grey line). **b**. Particle size distribution profiles for PDZ2 wild-type (black line) and mutant K214A/K250A (grey line) obtained by dynamic light scattering analysis (DLS). **c**. SEC-MALS analysis of the PDZ2 wild-type (black line) and K214A/K250A mutant (grey line) showing that both samples are characterize by a single specie of about 10 kDa.

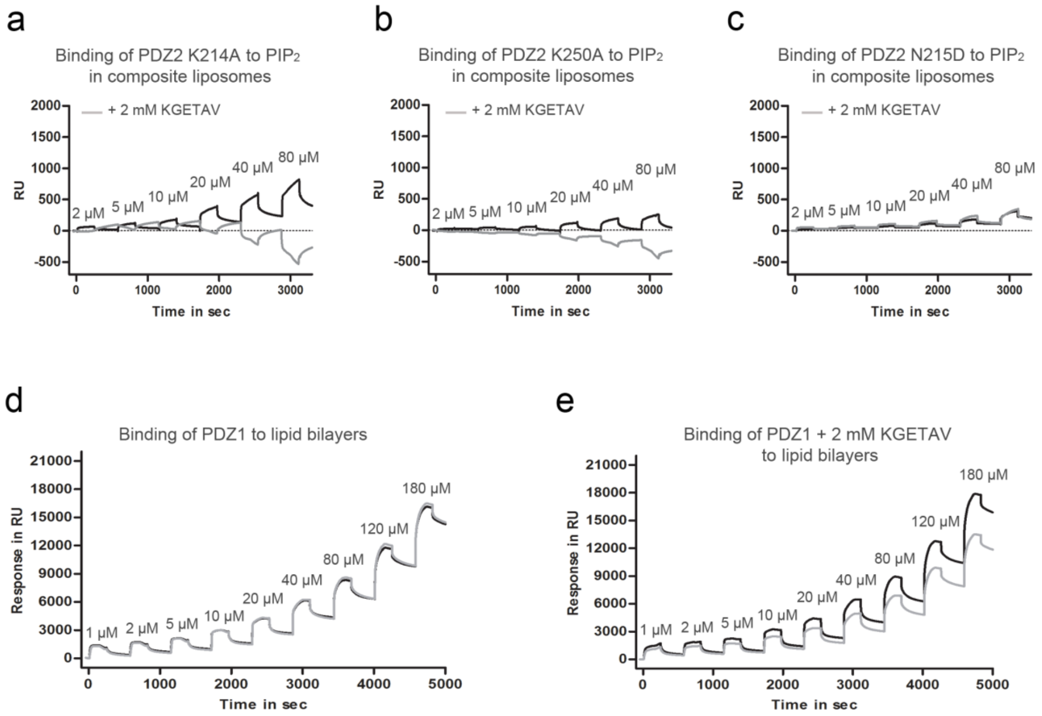

Supplementary Figure 3. The enhancement of PDZ2 interaction with PIP_2 embedded in liposomes in the presence of Frizzled 7 peptide is lost when the PDZ2 carries K214A, K250A or N215D mutations. PDZ1 in isolation strongly interacts with liposomes but shows poor if any interaction with PIP_2 embedded in liposomes. SPR sensorgrams illustrating the binding of PDZ2 K214A (**a**), PDZ2 K250A (**b**) and PDZ2 N215D (**c**) to liposomes containing 30% PC/ 40% PE/ 20% PS / 5% PI and 5% PIP_2 in the absence (black continuous line) or in the presence of 2 mM KGETAV Frizzled 7 peptide (grey continuous line) after substraction of the binding to control liposomes (30% PC/ 40% PE/ 20% PS / 10% PI). **d** and **e**. Sensorgrams illustrating the binding of increasing concentrations of PDZ1 wild-type to liposomes containing 30% PC/ 40% PE/ 20% PS and either 10% PI (black line) or 5% PI and 5% PIP_2 (grey line) in the absence (**d**) or in the presence (**e**) of 2 mM of KGETAV Frizzled 7 peptide. Sensorgrams are representative of three independent experiments.

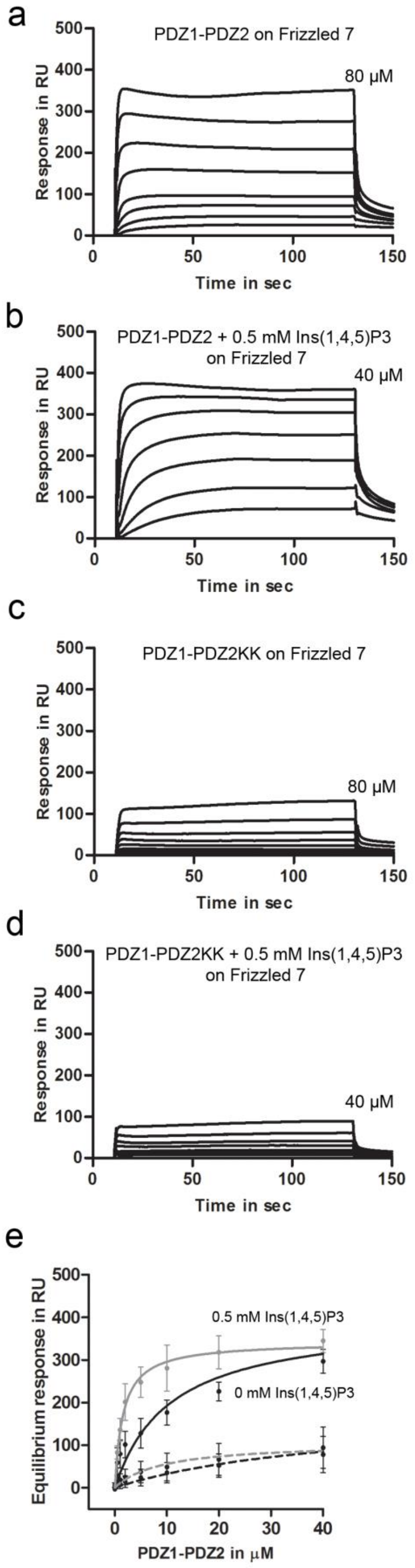

Supplementary Figure 4. SPR analysis of syntenin PDZ1-PDZ2 tandem-Frizzled 7 interaction. Blank reference-substracted SPR sensorgrams obtained for the binding of PDZ1-PDZ2 tandem wild-type (**a**) or PDZ1-PDZ2 tandem mutant K214A/K250A (**c**) (concentration ranging from 0.5 to 80 µM) to immobilized Frizzled 7 wild-type 19mer. To test the formation of the tripartite complexes, we perfused PDZ1-PDZ2 tandem wild-type (**b**) or PDZ1-PDZ2 tandem mutant K214A/K250A (**d**) with 0.5 mM Ins(1,4,5)P3 over immobilized Frizzled 7. **e**. Equilibrium binding isotherms of PDZ1-PDZ2 tandem wild-type (black continuous line) or PDZ1-PDZ2 tandem mutant K214A/K250A (black dotted line) in the absence or in the presence of 0.5 mM of Ins(1,4,5)P3 (grey continuous line for PDZ2 wild-type or grey dotted line for PDZ2 mutant K214A/K250A). Sensorgrams are representative of three independent experiments. Bars refer to ± s.d.

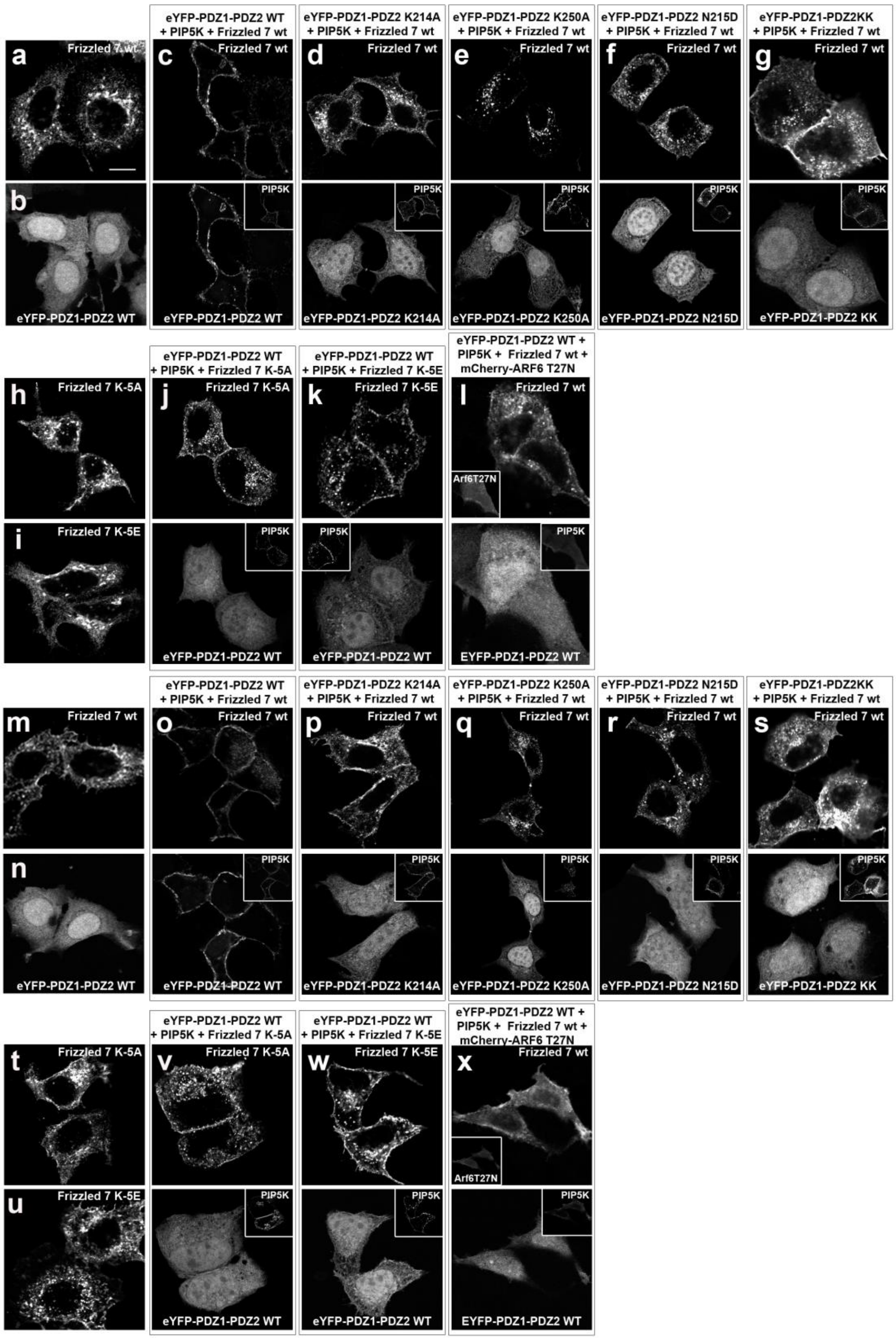

Supplementary Figure 5. Representative micrographs used for the quantification of the plasma membrane enrichment of Frizzled 7. MCF-7 cells were transiently transfected with expression vectors for Frizzled 7 wild-type, Frizzled 7 K-5A, Frizzled 7 K-5E, eYFP-PDZ1-PDZ2 tandem wild-type, eYFP-PDZ1-PDZ2 tandem K214A, eYFP-PDZ1-PDZ2 tandem K250A, eYFP-PDZ1-PDZ2 tandem N215D, eYFP-PDZ1-PDZ2 tandem K214A/K250A, phosphatidylinositol 4-phosphate 5-kinase (PIP5K) and/or mCherry-ARF6T27N as indicated. Frizzled 7 (wild-type or mutants) and PIP5K were detected by immunocytochemistry using labeled secondary antibodies and eYFP-PDZ1-PDZ2 tandem (wild-type or mutants) and mCherry-ARF6T27N were detected by direct fluorescence using confocal microscopy. Scale bar, 10 µm.

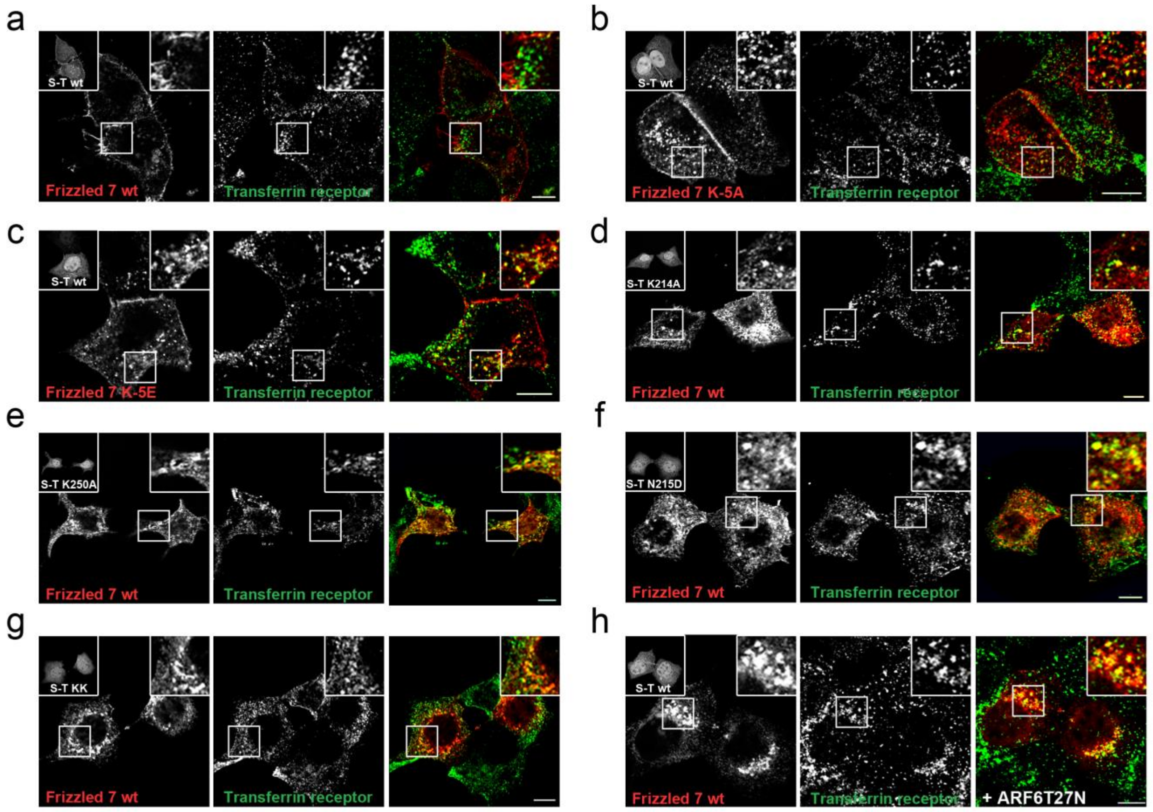

Supplementary Figure 6. Intracellular Frizzled 7 accumulates in the recycling compartment upon expression of syntenin or Frizzled 7 mutant defective for high affinity syntenin-Frizzled 7-PIP_2 binding (**a**-**h**). Representative confocal immunofluorescence micrographs of MCF-7 cells transiently transfected with expression vectors for Frizzled 7 wild-type or mutants (K-5A or K-5E), eYFP-PDZ1-PDZ2 tandem (S-T) wild-type or mutants (K214A, K250A, N215D or K214A/K250A) and ARF6T27N as indicated. Phosphatidylinositol 4-phosphate 5-kinase (PIP5K) is overexpressed in all panels (not shown). Cells were stained with anti-transferrin receptor antibody and appropriate secondary antibodies to label the recycling compartment. Note the partial but obvious co-localization of Frizzled 7 and the transferrin receptor in **b** to **h** when Frizzled 7 is mutated (**b**-**c**), eYFP-PDZ1-PDZ2 (S-T) is mutated (**d**-**g**) or when ARF6T27N is over-expressed (**h**). Scale bar, 10 µm

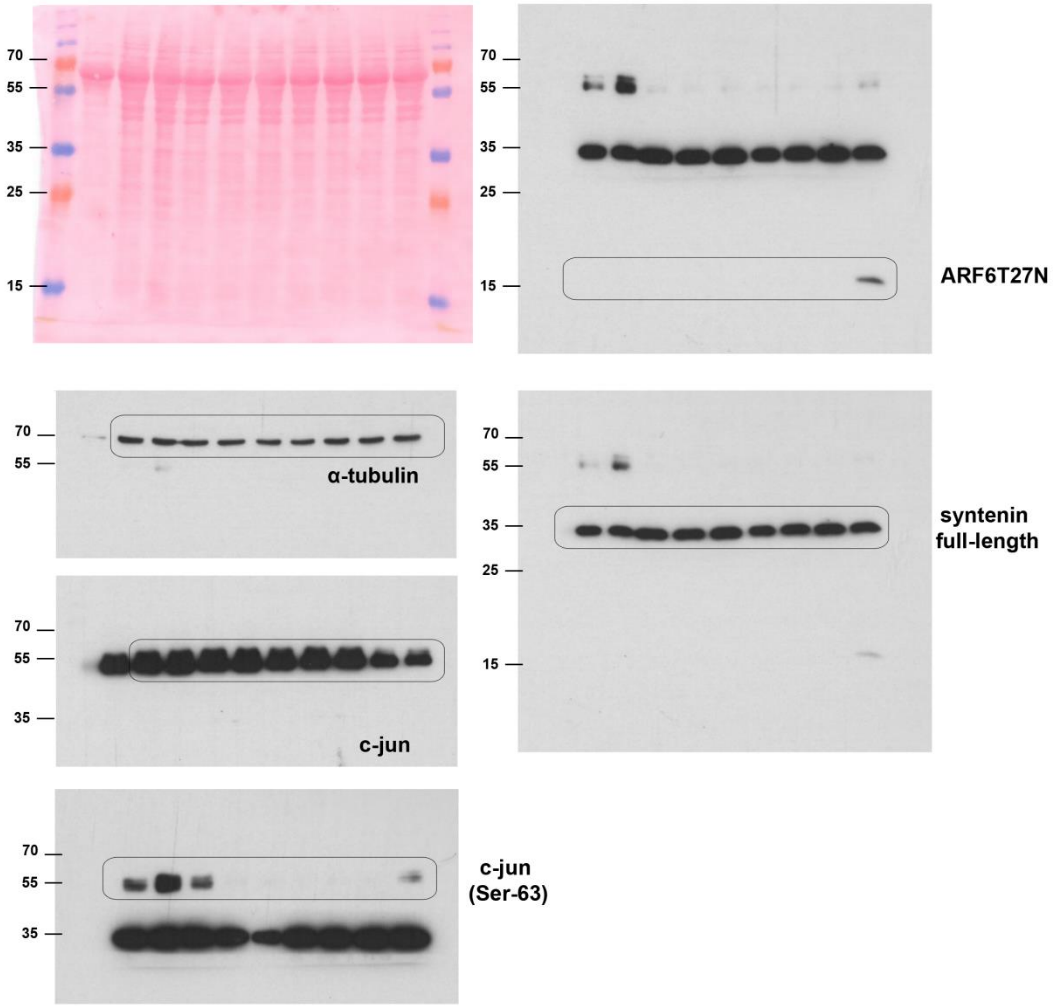

Supplementary Figure 7. Full scans of western blots.

Supplementary Table 1.

Thermodynamic analysis of the interaction of syntenin PDZ1-PDZ2 tandem with Frizzled 7 and Ins(1,4,5)P3 (the head group of PIP_2).

	**PDZ1-PDZ2 Frizzled 7 Site 1**	**PDZ1-PDZ2 Frizzled 7 Site 2**	**PDZ1-PDZ2 Ins(1,4,5)P_3**
N	1.28 ± 0.006	1.06 ± 0.017	0.99 ± 0.02
K (M^{-1})	9.08E4 ± 5.24E3	5.02E5 ± 9.98E4	4.1E4 ± 9.90E2
ΔH (Kcal/mol)	-9.74E3 ± 1.59E3	-3.66E4 ± 1.316E3	-1.10E3 ± 21.92
ΔS (cal/mol/deg)	-11.8 ± 5.66	-103 ± 5.02	17.2 ± 0.14

Values are ± s.e.m.

Supplementary Table 2.

Dissociation constants for the individual and simultaneous interactions of Frizzled 7 and Ins(1,4,5)P3 with syntenin PDZ1-PDZ2 tandem, as estimated by microscale thermophoresis.

	**PDZ1-PDZ 2* IP3**	**PDZ1-PDZ2 Frizzled 7***	**PDZ1-PDZ2 Frizzled 7* in presence of IP3**
K_D (µM)	6.17 ± 0.72	13.50 ± 0.23	1.16 ± 0.2

*This molecule was labeled for the assay. Values are ± s.e.m.
